# Invasion of a Legume Ecosystem Engineer in a Cold Biome Alters Plant Biodiversity

**DOI:** 10.3389/fpls.2018.00715

**Published:** 2018-06-05

**Authors:** Vanessa M. S. Vetter, Nils B. Tjaden, Anja Jaeschke, Constanze Buhk, Veronika Wahl, Pawel Wasowicz, Anke Jentsch

**Affiliations:** ^1^Disturbance Ecology, BayCEER, University of Bayreuth, Bayreuth, Germany; ^2^Biogeography, BayCEER, University of Bayreuth, Bayreuth, Germany; ^3^Geoecology, Physical Geography, University of Koblenz-Landau, Landau, Germany; ^4^Icelandic Institute of Natural History, Akureyri, Iceland

**Keywords:** disturbance, field experiment, high latitude invader, Maxent, plant community reorganization, sub-arctic climate, transformer species, vegetation dynamics

## Abstract

Plant ecosystem engineers are widely used to combat land degradation. However, the ability of those plants to modulate limiting abiotic and biotic resources of other species can cause damage to ecosystems in which they become invasive. Here, we use *Lupinus nootkatensis* as example to estimate and project the hazardous potential of nitrogen fixing herbaceous plants in a sub-polar oceanic climate. *L. nootkatensis* was introduced to Iceland in the 1940s to address erosion problems and foster reforestation, but subsequently became a high-latitude invader. In a local field survey, we quantified the impact of *L. nootkatensis* invasion at three different cover levels (0, 10–50, and 51–100%) upon native plant diversity, richness, and community composition of heath-, wood-, and grasslands using a pairwise comparison design and comparisons of means. Afterward, we scaled impacts up to the ecosystem and landscape level by relating occurrences of *L. nootkatensis* to environmental and human-mediated variables across Iceland using a species distribution model. Plant diversity was significantly deteriorated under high lupine cover levels of the heath- and woodland, but not in the grassland. Plant species richness of the most diverse habitat, the heathland, linearly decreased with lupine cover level. The abundance of small rosettes, cushion plants, orchids, and small woody long-lived plants of the heath declined with invader presence, while the abundance of late successional species and widespread nitrophilous ruderals in wood- and grasslands increased. Distribution modeling revealed 13.3% of Iceland’s land surface area to be suitable lupine habitat. Until 2061–2080, this area will more than double and expand significantly into the Central Highlands due to human mediation and increasingly favorable climatic conditions. Species-rich habitats showed a loss of plant species diversity and richness as well as a change in community composition even in low lupine cover classes. The future increase of suitable lupine habitat might lead to the displacement of cold-adapted native plant species and will certainly challenge conservation as well as restoration of ecosystems in the cold climate of Iceland, but also elsewhere. Lupine invasion speeds up succession, which may be additive with climate change effects, and accelerates ecological change in cold biomes.

## Introduction

Invasive plants are globally threatening ecosystems and island floras leading to species endangerment and extinction (Pejchar and Mooney, 2009; Harter et al., 2015). Especially invasive ecosystem engineers can strongly influence native ecosystems by altering energy, water and/or nutrient fluxes, which consequently leads to altered ecosystem-level properties (e.g., *Myrica faya* a nitrogen fixing tree invasive in Hawaii; Vitousek et al., 1987; Vitousek and Walker, 1989). Ecosystem engineers (Jones et al., 1994) are often intentionally introduced to new environments by humans, e.g., for soil and water conservation, to restore degraded ecosystems or to solve the problems of deforestation (Lazzaro et al., 2014; Ayanu et al., 2015). They generally possess traits that can positively influence soil stability, nutrient and hydrological cycling, and light infiltration (Ayanu et al., 2015) and show protective characteristics, e.g., reduced erosion (Fei et al., 2014). But if they become invasive, those positive traits of the respective alien ecosystem engineer can have negative and long-lasting effects on native communities and ecosystem properties (Richardson et al., 2000; Catford et al., 2012; Fei et al., 2014) that often extend far beyond its life span and/or presence (Richardson et al., 2000; Ehrenfeld, 2003, 2010). Ecosystem engineers that have become invasive, are called “transformer species” (Richardson et al., 2000). Invaders that are introduced for management purposes, such as the ecosystem engineers, are usually widely and deliberately applied by humans and are thus able to spread into large areas right at the beginning of the invasion process with many starting points for the invasion.

*Lupinus nootkatensis* DONN ex SIMS acts as an ecosystem engineer in the sub-polar ecosystems of its invasive range Iceland. Originally from Alaska and Canada, this high-latitude invader was introduced to Iceland in 1945 for soil amelioration and reforestation. Due to repeated human introductions, *L. nootkatensis* has a high propagule pressure and is rapidly spreading across the Icelandic lowlands (Magnusson, 2010). *L. nootkatensis* stabilizes slopes and modulates limiting abiotic resources of other species by fixing atmospheric nitrogen, thus changing the nutrient cycling of invaded habitats. Cold biomes show a rapid saturation in the ecosystem’s capacity to retain N, making them prone to N_2_ fixers (Hiltbrunner et al., 2014). Such changes caused by the accumulation of atmospheric nitrogen in the soil and subsequently in the plant community composition are persistent and continue even after the removal of the legume from the ecosystem or its replacement by other species (Hiltbrunner et al., 2014). The increased soil nitrogen content in old lupine stands facilitates the settlement of further invasive species, such as demonstrated for, e.g., *Anthriscus sylvestris* and *Ribes rubrum* in Iceland (Magnusson et al., 2008; Magnusson, 2010). *L. nootkatensis* modulates biotic factors such as plant–plant interactions by forming dense patches, affecting plant establishment and succession of arctic plant species via direct competition effects (Magnusson et al., 2008; Magnusson, 2010). *L. nootkatensis* is also a habitat generalist, and widely occurs across Icelandic lowland habitats (Magnusson, 2010). It transforms the native vegetation, e.g., heathlands, into *Poa pratensis* dominated grasslands (Magnusson et al., 2008), thus directly affecting plant establishment and succession. However, *L. nootkatensis’* ability to facilitate soil enrichment and succession, by building up nutrients, organic matter, and water storage capacity of soils is perceived as one solution to combat the manmade and massive problem of severe land degradation and desertification in Iceland (Arnalds and Runolfsson, 2008), which also may be exacerbated by future climate change.

The combination of species invasion and climate change might lead to negative synergistic effects, which are more powerful than the additive effects of the two single stressors. Despite the buffering effects of the surrounding oceans, climate change will lead to profound alterations of the environmental conditions on islands, which might positively affect the establishment and spread of alien species in various ways (Harter et al., 2015).

We investigate lupine invasions in different plant communities on a local scale field study and scale up to the ecosystem and landscape level using a correlation model. It is currently under debate which factors are mainly responsible for the ecosystem engineer’s ongoing spread in Iceland and how climate change will affect these factors in the future. Although, there are existing studies concerning the community impact, the invasion success and the future distribution of *L. nootkatensis* in Iceland, most of these studies only concern one or few factors of the same kind, e.g., different climate variables or biotic interactions. Here, we set out to address and quantify the relative influence of a variety of abiotic, biotic and human-mediated factors, which are probably determining the actual distribution pattern of *L. nootkatensis* across Iceland and project the likelihood of lupine-free areas to become invaded in the near future. The rapid spread, ability to alter its local environment, and its habitat generality make *L. nootkatensis* an interesting case study for invasion processes in cold biomes, e.g., the consequences of exotic invasion in niche construction (Fei et al., 2014). Combining experimental studies of local communities with predictive modeling at a landscape level, provides a more accurate overview of the potential range of the species in Iceland (Stricker et al., 2015). The spatially enclosed system of Iceland is well-suited for our approach because of its insularity, the excessive spread of *L. nootkatensis* into a great variety of plant communities of the Icelandic lowlands and the relatively precise documentation of its introduction into the sub-polar system (Magnusson et al., 2008; Magnusson, 2010).

We aim to (a) quantify the current invasion status of *L. nootkatensis* on Iceland using a distribution map of the year 2016, (b) quantify the invasion impacts of the ecosystem engineer on the native vegetation (hereafter: biotic characteristic) in Iceland, (c) understand the abiotic and biotic filters decisive for the recent invasion success, and (d) robustly project the invasion range of *L. nootkatensis* in Iceland under current (reference period: 1960–1990) and future (2061–2080) climate conditions based on the findings of a and b. We use two distinct data sets: (1) a field study to test the biotic characteristics and (2) a distribution map to model the abiotic characteristics as well as the invasion process.

## Materials and Methods

### Study Species

*Lupinus nootkatensis* (Fabaceae) is a long-lived (up to 20 years) herbaceous plant originating from coastal regions of the Aleutian Islands and from Queen Charlotte Island, Alaska to Vancouver Island, British Columbia, Canada (Magnusson, 2010). *L. nootkatensis* prefers open habitats of frequent natural disturbance (Fremstad and Elven, 2008), e.g., early successional stages with vegetation destruction and soil erosion. In Iceland, the lupine is primarily recorded from gravel bars along the coast and rivers, roadsides, dry slopes and sandy beaches. But it is also found in disturbed landscapes, as well as in dwarf shrub-heathlands (Magnusson, 2010).

### Biotic Filter Experiment and Propagule Pressure

#### Study Area

**FIGURE 1:**
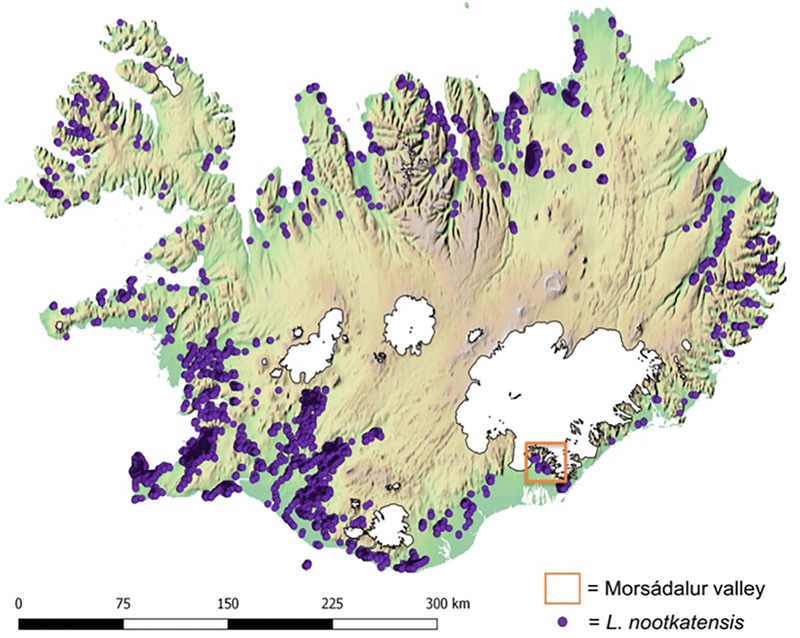
Distribution of *Lupinus nootkatensis* across Iceland in 2016. Lupine occurrences (Icelandic Institute of Natural History http://en.ni.is/) are displayed upon an altitudinal gradient where green indicates the lower and brown the higher regions of Iceland and glaciers are displayed in white. Our study area, the Morsádalur valley in South-East Iceland, is located in the Vatnajökull National Park.

The study area of the local field survey, Morsádalur, is located in the Vatnajökull National Park in South-East Iceland (**Figure 1**). The Vatnajökull area is greatly influenced by glacial and volcanic processes (Steinthorsson et al., 2000; Björnsson, 2003; Björnsson and Pálsson, 2008). Within the sub-polar oceanic climate of Iceland, the valley Morsádalur is characterized by a mild climate with warm temperatures (Björnsson et al., 2007) and high annual precipitation (Crochet et al., 2007; Björnsson and Pálsson, 2008). We chose three different habitat types, which are characteristic for the native vegetation of Iceland and most dominant, and are currently invaded by *L. nootkatensis*: a heathland on the mountain slope Réttargíl, a grassland with occasional trees in the valley Morsádalur and the old birch forest (*Betula pubescens*) Bæjarstadarskógur on the adjacent western slope of Morsádalur.

#### Sampling Design and Methods

To test the effect of lupine invasion on plant community composition among three different habitats a pairwise comparison design, between the cover levels within and among each habitat type, was employed.

First, we defined three different levels of lupine cover density: “none,” which had no lupines in the vegetation cover, “low” which had 10–50% lupine cover, and “high” which had 51–100% lupine cover (Magnusson et al., 2008). Areas with 1–9% of lupine cover were excluded from the analysis because these areas are mainly occupied by immature lupine plants. This gradient in lupine invasion succession was observed along transects from the center to the edge of a lupine patch. While the center represents late invasion stages with high lupine cover, the edges of a lupine patch represent early invasion stages with relatively low lupine cover (Magnusson et al., 2008).

Three plots of 2 m × 2 m size for each of the three lupine cover density levels were randomly assigned to the lupine patches of each habitat (in total = 27 which consist of 3 × 3 = 9 plots per habitat). The plot size of 2 m × 2 m was determined by a minimum area analysis to cope with the heterogeneity of the habitats and represents the largest minimum area found in the heathland. Plots of the same density level were not placed within the same lupine patch, although where possible, different density levels did occur within the same patch.

Soil seed bank of *L. nootkatensis* was estimated by taking one soil core of 5 cm diameter and depth per plot. Thus, soil samples were replicated three times per cover level of each habitat (*n* = 27). All soil samples were taken at the end of the field season in August within one single day. For levels “low” (10–50%) and “high density” (51–100%) the core was randomly taken at 40 cm distance to the lupine chosen as reference for the nearest neighbor analysis. Samples were air-dried and sieved through three sieves with mesh sizes of 16, 8, and 4 mm. We sorted and counted the lupine seeds by hand.

We additionally analyzed plant community composition and nearest neighbor growth patterns of the three habitats to pinpoint potential differences between lupine cover levels (see Appendix Figures A1–A3).

#### Statistical Analyses

As a measure of alpha diversity within habitats and plots the Simpson (diversity) index, also called Simpson concentration, was calculated separately for each of the three plots per lupine cover level and habitat (Simpson, 1949; Lande, 1996):

$$\lambda =\sum_{i=1}^{R}{\text{p}}_{\text{i}}^{\text{2}}$$

R is the richness of each habitat type, p_i_ is the squared relative abundance of the respective species and λ is the probability of two randomly chosen specimen to belong to the same species. Thus, a Simpson index of 0 represents highest diversity, while a value of 1 represents no diversity.

Analyses were conducted using the statistical software R 3.4.2 (R Core Team, 2017). The effects of habitat and lupine cover level within habitats on the alpha diversity, plant species richness, seed abundance and soil depth were tested via ANOVA and *post hoc* Tukey-test in case of normally distributed data with variance homogeneity (Hothorn et al., 2008). The Kruskal–Wallis test for multiple comparisons (Giraudoux, 2017) was applied to data with an inhomogeneous variance or residuals that did not follow the normal distribution. We used the Bartlett-test and the Shapiro–Wilk test to check for variance homogeneity among the groups and normal distribution of the residuals respectively.

### Modeling the Spatial Distribution of *L. nootkatensis* in Iceland

We used the model algorithm Maxent (Phillips et al., 2017) version 3.4.1 to relate occurrences of *L. nootkatensis* to environmental variables across whole Iceland.

#### Species Occurrence Data and Environmental Variables

Abiotic, biotic and human-mediated environmental variables, which are associated with the range limits of *L. nootkatensis* in Iceland according to literature, were pre-selected by expert knowledge (**Table 1**) to determine the most influential variables.

**Table 1:** Environmental predictor variables pre-selected by expert knowledge.

Category	Variables	Source	Reference
Climate data	Annual mean temperature, temperature seasonality, **maximum temperature of warmest month**, minimum temperature of coldest month, minimum temperature of May, **mean temperature of wettest quarter**, mean temperature of warmest quarter, annual precipitation, precipitation of driest month, **precipitation seasonality**, precipitation of wettest quarter, precipitation of driest quarter, precipitation of warmest quarter	Bioclimatic variables WorldClim1.4 – Global Climate Data of the current (reference period 1960–1990) climate conditions. Bioclimatic variables for future climate scenarios (CMIP5): NorESM1-M (RCP 4.5, RCP 8.5) MPI_ESM-LR (RCP 4.5, RCP 8.5) (Hijmans et al., 2005)	Magnusson et al., 2008; Magnusson, 2010; Wasowicz et al., 2013

Topography	Altitude	Bioclimatic variables WorldClim1.4 – Global Climate Data (Hijmans et al., 2005).	Own consideration in accordance with Magnusson, 2010
	Aspect and slope	Manually calculated from altitude in R	

Soil	Age of substrate	Icelandic Institute of Natural History (http://en.ni.is/). Accessed October 17, 2016.	Own consideration in accordance with Sigurdardottir, 2008; Magnusson, 2010
	Soil type	Agricultural University of Iceland (provided February 27, 2018)	Personal communication Dr. Olafur Arnalds; Arnalds, 2015

Land cover	**Vegetation types:** grassland and cultivated land, birch woodland, moss heathland	Icelandic Institute of Natural History (http://en.ni.is/). Accessed October 17, 2016.	Hultén, 1968; Fremstad and Elven, 2008;
	**Surface water:** rivers, waterbodies, glaciers		Magnusson, 2010
	**Substrate:** sand, lava, gravel plains		

Human vector	**Distance to nearest road** **Human influence index** (human population pressure; human land use and infrastructure; human access)	Calculated based on the road map obtained from the NLSI: National Land Survey of Iceland (http://www.lmi.is/en/). Accessed January 04, 2017. Wildlife Conservation Society – WCS, and Center for International Earth Science Information Network – CIESIN – Columbia University, 2005. Last of the Wild Project, Version 2, 2005 (LWP-2). Palisades, NY: NASA SEDAC. doi: 10.7927/H4BP 00QC. Accessed January 04, 2017.	Magnusson, 2010

*Variables in bold were further selected by Pearson Correlation Coefficient, Jackknife and AIC and used to calibrate the species distribution model 1 (Maxent) of *Lupinus nootkatensis*. In model 2, we omitted the variable “distance to nearest road” but kept all other settings constant.*

We used climate data together with characteristics of the terrain (e.g., aspect and slope), soil type, geology, native vegetation cover, and aspects of human interference (**Table 1**) as a proxy to test how much of Iceland’s land surface area is threatened by lupine invasion. Aspect and slope in combination with the climate variables control for the self-propagation of the invader species (Magnusson, 2010), while all other variables are potential factors determining the distributional range of *L. nootkatensis* (see e.g., Magnusson et al., 2008; Magnusson, 2010; Wasowicz et al., 2013).

Climate data for current and future conditions was obtained from Worldclim 1.4 (Hijmans et al., 2005) at a spatial resolution of 30 arc seconds (≈1 km). To predict the potential future distribution of the legume invader in Iceland, downscaled and calibrated climate data from the global climate models (GCM) NorESM1-M and MPI_ESM-LR for the years 2061–2080 was used. Both, the medium stabilization (RCP 4.5) (Thomson et al., 2011) and very high baseline emission (RCP 8.5) (Riahi et al., 2011) representative concentration pathways of the IPCC’s fifth assessment report were used.

If necessary, other variables were projected to WGS84, rasterized and re-sampled (Hijmans, 2016) to the 1 km spatial resolution of the climate variables.

The species occurrence data was obtained from the Icelandic Institute of Natural History^1^ in the form of spatial polygons representing *L. nootkatensis* patches derived from high-resolution satellite imagery. We converted the spatial polygons to a raster of the same spatial resolution and dimensions as the environmental data using the “rasterize” function in R (Hijmans, 2016). The center point of each grid cell containing *L. nootkatensis* patches were then used to derive the needed occurrence records for Maxent. A total of 5709 species occurrences were compiled across Iceland (**Figure 1**).

We used the open source software R version 3.4.2 (R Core Team, 2017) and QGIS 2.16.3 in order to prepare the species occurrence records as well as all environmental variables (background data) as spatial data layers.

#### Species Distribution Model

We calculated Pearson correlation coefficients (r) in R to derive a set of fairly uncorrelated environmental variables. Because Maxent copes well with collinearity (Elith et al., 2011), cross-correlation was used as a selection criterion only to exclude the highest correlative variables (*r* > 0.8).

The remaining variables were used to calculate principal component analyses (PCAs) based on which we measured spatial heterogeneity of the environment. The derived grids of environmental heterogeneity were then used to spatially rarefy our species occurrence points (“Spatially Rarefy Occurrence Data for SDMs” tool, SDMtoolbox; Brown, 2014). Overall, 98 unbiased species occurrences were used in Maxent.

For invasive species, the absence of occurrences means not necessarily a “true absence” due to, e.g., the unsuitability of the respective habitat, but rather a reflectance of the fact that the species has not yet reached its equilibrium distribution in the new habitat. Therefore, we treated our species data as presence-only data. Maxent is a common and very effective methodology to model the ecological niche of species with presence-only data (Elith et al., 2006; VanDerWal et al., 2009; Phillips et al., 2017) but it needs to be provided with a set of background data (VanDerWal et al., 2009; Barve et al., 2011). As the dispersal potential of the invasive species might be large, e.g., due to human traffic or targeted propagation by humans, we opted for a buffer-based approach for background sampling. Following the example of VanDerWal et al. (2009), we produced a series of test models using buffer zones with radii of 1 km (size of one raster grid cell), 5, 10, 25, and 50 km. In our case, a buffer zone with a radius of 25 km gave the best result.

Jackknife testing within Maxent along with the Akaike Information Criterion (AIC) implemented in R, were used to select the final environmental variables for the species distribution model (model 1, **Table 1**). We gradually removed all variables whose regularized training gain was less than 0.1, unless the AIC and AUC of model 1 significantly deteriorated. To evaluate model performance, we ran a 10-fold cross-validation (cv) after each simplification.

The ENMeval package in R (Muscarella et al., 2014) was used to tune Maxent settings, as well as for model validation. We tested regularization multiplier (RM) values of 1, 2, 5, 10, 15, 20 (Warren and Seifert, 2011; Merow et al., 2013; Shcheglovitova and Anderson, 2013) together with different combinations of the Maxent feature classes linear (L), quadratic (Q), and hinge (H) (Merow et al., 2013; Phillips et al., 2017) with block-wise data partitioning (Roberts et al., 2017).

We fitted two final models using all of the spatially rarefied species occurrences, RM = 5, LH features, and a maximum of 1000 iterations. The cloglog output format was chosen for both models (Phillips et al., 2017). Model 1 was used to evaluate the environmental variables decisive for the actual spread pattern as well as to predict the potential distribution of *L. nootkatensis* across Iceland under current and future climate conditions. To evaluate the potential maximum area of suitable habitat available for *L. nootkatensis* under current and future climate conditions, without the restriction to roads as the vectors of propagation, we fitted a second model and calculated difference maps based on the predictions of both models (see Appendix). Model 2 was fitted with the same settings as model 1, but without the variable distance to nearest road.

The cloglog output format gives probabilities of occurrences for the respective species varying between 0 and 1. We used the maximum training sensitivity plus specificity threshold, a threshold selection method which is not affected by pseudo-absences (Liu et al., 2013), to reclassify the cloglog output in suitable (>threshold) and unsuitable habitat (<threshold).

To assess the accuracy of our species distribution model we calculated partial receiver operating characteristics (Peterson et al., 2008; Tjaden et al., 2017) with 1000 bootstrapping iterations on 50% of the test data and an expected error rate of 5%.

## Results

### Biotic Filter Experiment and Propagule Pressure

**FIGURE 2:**
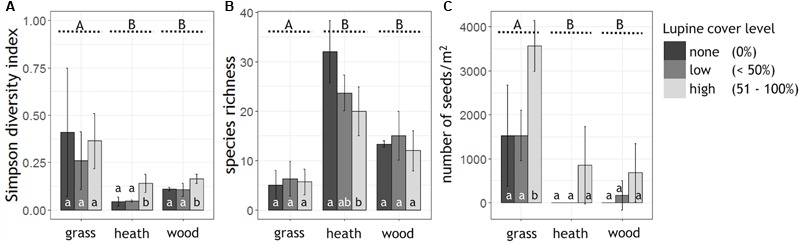
Alpha diversity, species richness and seed abundance grouped by habitat and shown as a function of lupine cover level. Shown are the mean values of the **(A)** Simpson diversity index (0 = highest diversity, 1 = no diversity) and **(B)** plant species richness per lupine cover level and habitat (n_grass_ = 9, n_heath_ = 9, n_wood_ = 9). **(C)** Extrapolated lupine seed numbers per m^2^ (n_grass_ = 3, n_heath_ = 3, n_wood_ = 3). Capital letters A, B indicate significant differences between the habitats (Kruskal–Wallis test, *p* < 0.05, black dashed lines), while small letters a, b, indicate significant differences between the lupine cover levels within one habitat (Tukey HSD or Kruskal–Wallis test, *p* < 0.05).

High lupine cover levels significantly reduced the alpha diversity of the heath- and woodland (Simpson diversity index; **Figure 2A**). In the grassland, lupine cover did not have a significant effect on alpha diversity. Plant species richness of the heathland, the most diverse habitat, decreased linearly with lupine cover level (**Figure 2B**). In the woodland as well as in the grassland, species richness showed a slightly hump-shaped pattern from none to high lupine cover.

Typical heath species such as *Calluna vulgaris, Empetrum nigrum*, and *Arctostaphylos uva-ursi* decreased in their abundance with proceeding lupine invasion. The percentage cover of *Calluna vulgaris* was halved in both, low and high cover classes of *L. nootkatensis* (Appendix Figure A1). Small rosettes (*Silene acaulis*), cushion plants (*Armeria maritima*), orchids (*Listera cordata, Dactylorhiza maculata, Platanthera hyperborea*) and small woody long-lived plants (e.g., *Salix herbacea*) of the heathland were absent in the presence of the invader, even in low lupine cover classes (Appendix Table A1). In the heathland as well as in the woodland, the abundance of late successional species, e.g., *Betula pubescens*, increased with lupine cover (Appendix Figure A1). In high lupine cover classes widespread nitrophilous plants – *Taraxacum* sp. in the woodland and *Geranium sylvaticum* in the grassland – appeared, while they were not present in low cover classes or without the invader (Appendix Table A1). *Poa pratensis*, the most abundant grass in the grassland decreased remarkably, while a contrasting trend was observed for *Angelica archangelica*, another late successional species (Appendix Figure A1).

Abundance of *L. nootkatensis* seeds in the soil differed significantly among habitats. The most diverse habitat, the heathland, had the lowest abundance of lupine seeds while the least diverse habitat, the grassland, showed highest seed numbers (**Figure 2C**). Propagule pressure of lupine seeds tended to be highest in patches with 51–100% lupine cover while it was indifferent in the cover classes “none” and “low,” although this effect was only statistically significant in the grassland but not in the other two habitat types. Only in the woodland, the expected trend toward no seeds without lupine cover, few seeds with low lupine cover and increased seed abundance in high lupine cover stands was observed (**Figure 2C**).

### Modeling the Spatial Distribution of *L. nootkatensis* in Iceland

Both Maxent models had a good predictive ability as measured by the area under the curve (AUC_model1_ = 0.84, AUC_model2_ = 0.79) and the AUC ratios of the partial receiver operating characteristics (mean AUCratio_model1_ = 1.76, mean AUCratio_model2_ = 1.70). All values ≥ 0.531 and 0.553 respectively (maximum training sensitivity plus specificity threshold) were interpreted as suitable lupine habitat. The five most important variables influencing the distribution of *L. nootkatensis* across Iceland were distance to nearest road, maximum temperature of warmest month, land cover, mean temperature of wettest quarter, and precipitation seasonality (**Table 2**).

**Table 2:** Percent contribution and permutation importance of the environmental variables used in the final models.

Predictor variable	Contribution [%]	Permutation importance
Distance to nearest road	72.3 (–)	53.4 (–)
Maximum temperature of warmest month	12.1 (52.6)	24.3 (54.7)
Land cover	6.3 (22.8)	5.0 (7.5)
Mean temperature of wettest quarter	5.6 (13.1)	14.6 (22.0)
Precipitation seasonality	3.7 (10.0)	2.7 (15.6)
Human influence index	0 (1.3)	0 (0.2)

*Results of model 2 are given in brackets. The higher the relative information of a single variable, the more decisive it is for the current pattern of propagation.*

**FIGURE 3:**
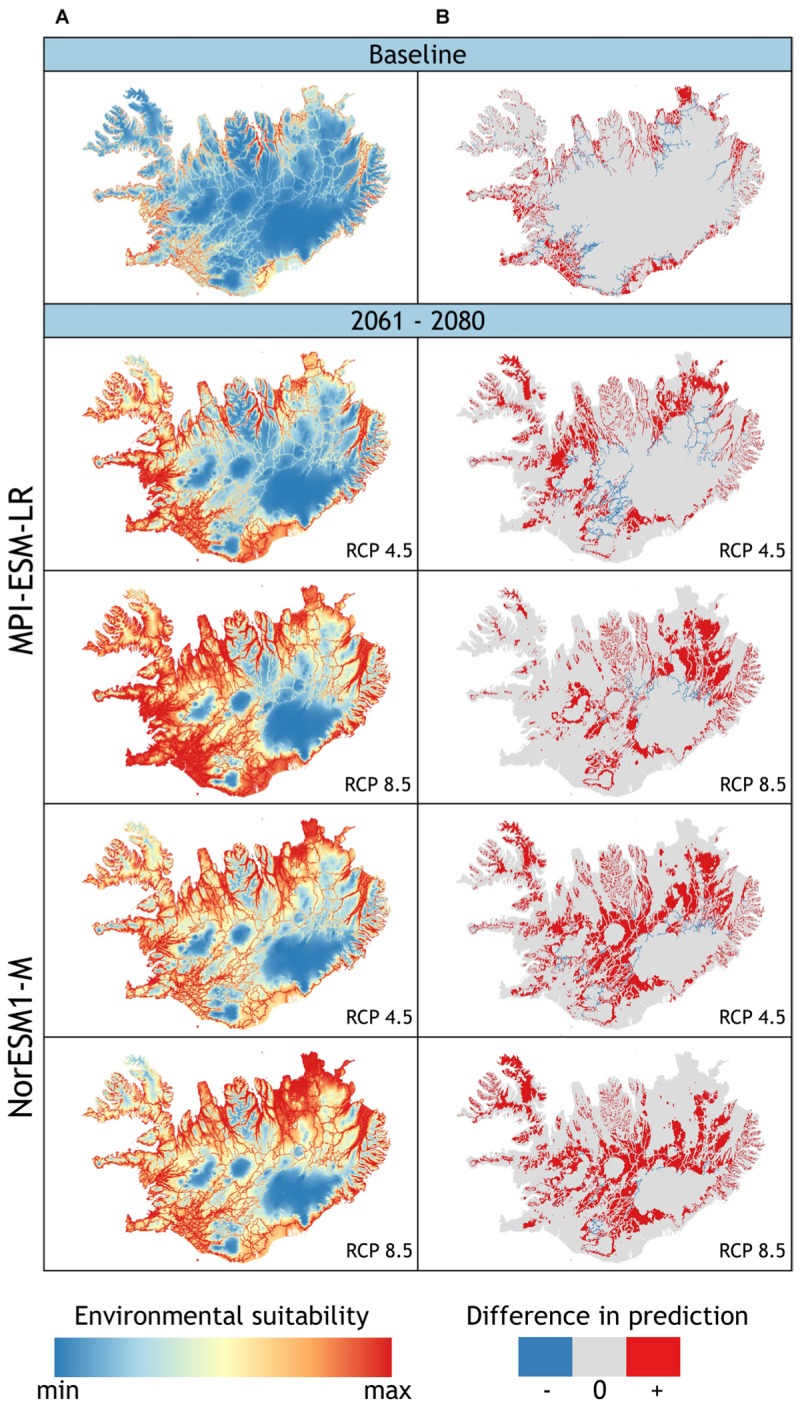
**(A)** Projected potential distribution (model 1) of *L. nootkatensis* across Iceland under current climate conditions (baseline) and future climate conditions modeled with the global climate models NorESM1-M and MPI_ESM-LR each in the medium stabilization (RCP 4.5) and very high baseline emission scenario (RCP 8.5). Environmental suitability ranges from: minimum = 0 to maximum = 1 with maximum training sensitivity plus specificity threshold (MTSS) = 0.531. **(B)** Difference in prediction between the two models used to project *L. nootkaensis’* potential future distribution [difference = binary output (model 2 – model 1)] (see Appendix). The models only differ in the presence of the propagation vector “distance to nearest road”: model 1 vector present, model 2 absent. Areas projected to be suitable habitat only by model 2, shown in red, are an addition to the projections of model 1 (+); areas in gray (0) are equally projected from both models, while areas in blue (–) are solely projected by model 1. Together with the projections of model 1, red areas show the maximum of suitable lupine habitat without restriction to “roads” as the vectors of propagation.

Under current climate conditions, a total of 13.3% of Iceland’s land surface area was projected to be suitable lupine habitat (**Figure 3** and **Table 3**). *L. nootkatensis* was mainly found in habitats close to roads (≤0.5 km). The predicted probability of presence shows an optimum at 14.4°C for the maximum temperature of the warmest month, at 8.2°C for the mean temperature of the wettest quarter, and at 2.7 for the precipitation seasonality (Appendix Figure A4). *L. nootkatensis* was found in all land cover classes across Iceland, but the invasion risk was projected to be highest for grassland/cultivated land and lowest for moss heath and wetlands (Appendix Figure A4).

**Table 3:** Percentage amount of land surface area of Iceland projected to be suitable habitat for *L. nootkatensis* under current and future climate conditions.

Time scale	Concentration pathway	Projected amount of suitable habitat [%] (% increase compared to current)	
		Model 1	Model 2
Current	–	13.3	20.1
MPI_ESM-LR	RCP 4.5	39.1 (+ 25.8)	53.2 (+ 33.1)
	RCP 8.5	61.7 (+ 48.4)	76.7 (+ 56.6)
NorESM1-M	RCP 4.5	50.1 (+ 36.8)	72.6 (+ 52.5)
	RCP 8.5	58.0 (+ 44.7)	81.2 (+ 61.1)

*The percentage point increase in suitable habitat compared to current climate conditions is given in brackets.*

Independent from the tested emission scenarios both GCMs projected a more than double increase in the amount of suitable lupine habitat for the years 2061–2080 (**Table 3**). With proceeding climate change, the environmental suitability of Iceland was projected to expand into the Central Highlands, thus the potential distribution range of *L. nootkatensis* will enlarge. For example, in 2016 *L. nootkatensis* occurred on altitudes up to 572 m but was projected to reach heights of 1087–1119 m (MPI_ESM-LR RCP 8.5, NorESM1-M RCP 8.5) in the future. *L. nootkatensis* is likely to spread from its current main distribution area – along the coasts and near the human settlements – following the main valleys and roads into the Central Highlands. While roads serve as vectors of propagation (model 1, **Figure 3A**), *L. nootkatensis’* occurrence is not dependent upon the presence of roads under future climate conditions (**Figure 3B**). The future spatial focus was projected to lie in the northern to northeastern and southwestern parts of the island.

## Discussion

The restrictive factor(s) controlling lupine colonization is depending on the respective area. Low propagule pressure, is impeding lupine spread in areas without major human interference, e.g., the highlands. *L. nootkatensis* is a very effective disperser, in terms of durability and amount of produced seeds, and in addition its spread is accelerated by human interference (Magnusson, 2010; Wasowicz, 2016). We detected a large quantity of seeds even in areas where currently no or only a few lupines are growing. The large amount of seeds in the rather low lupine cover classes of the grasslands are either deposited by the river Morsá which floods the valley of Morsádalur at irregular intervals – an important avenue for propagule dispersal facilitated by frequent disturbance dynamics (Magnusson, 2010) – or are part of an old seed bank (Svavarsdóttir et al., 2008). Additionally, our results imply, that a high plant species diversity seems to go along with a lower overall seed abundance of *L. nootkatensis*, potentially reducing risk of invasion. However, in high lupine cover levels the natural diversity decreases and the invader is able to build up persistent seed banks. The missing significance of these results might be due to the extreme patchiness of seed banks. Increasing the number of soil samples per plot could overcome this obstacle. Consequently, propagule pressure, one of the key drivers and a prerequisite for successful invasion (Lockwood et al., 2005; Colautti et al., 2006; Catford et al., 2009) is not limiting but delaying *L. nootkatensis’* distribution across the highlands and mountainous areas of Iceland. Biotic competition in areas void of disturbance such as the grassland, seems to impede colonization of *L. nootkatensis*, however, as the lupine seeds are durable, it is only a matter of time until disturbance occurs and colonization is facilitated (Sigurdsson and Magnusson, 2008). Abandonment of traditional management practices, e.g., free-ranging sheep grazing, might further facilitate lupine establishment as sheep graze on small seedlings and thus prevent the lupine from establishing (Magnusson et al., 2008). Based on the SDM projections, sheep grazing could now systematically be used to restrict the predicted potential distribution of *L. nootkatensis* across Iceland, while maintaining a traditional management system.

Our results suggest that *L. nootkatensis* may benefit from anthropogenic influences, though is not necessarily dependent on human presence. Initially, areas close to human infrastructure (e.g., roads) are exposed to a higher invasion risk, but as the invasion progresses, the lupine increasingly decouples from the roads as primary vectors of propagation and begins to penetrate large areas of the Central Highlands. Since propagule pressure increases with time and due to *L. nootkatensis’* long residence time in Iceland, starting with its first introduction in 1945 (Magnusson, 2010), seed swamping around human settlements can be assumed (Colautti et al., 2006; Catford et al., 2009). Human-mediated disturbance along with sufficient propagule pressure creates invasion windows as disturbances reduce competition, increase space and subsequently resource availability (Catford et al., 2009). Based on our results we are able to verify the recently postulated relation between human disturbance and occurrence of invasive species (Wasowicz, 2016) for *L. nootkatensis*.

All current hot-spots of invasive plant species occurrences in the Central Highlands are linked to human disturbance, e.g., tourism and the related infrastructure (Wasowicz, 2016). Tourism in general but also the number of visitors of the Icelandic highlands is sharply increasing in recent years (Icelandic Tourist Board, 2017). Thus, one of the last wilderness areas of Europe (Sæórsdóttir and Saarinen, 2015) becomes gradually more accessible for propagules and at the same time more disturbed by human visitors (Wasowicz, 2016).

Arctic and sub-arctic regions will be affected by climate change in a twofold way: (1) the cold-adapted native plants will be expelled and forced to migrate with their shifting climatic niche, e.g., upwards or northwards (Phoenix and Lee, 2004; Parmesan, 2006), (2) due to the temperature increase the (sub-) arctic regions will become more and more accessible to alien plants (Crumpacker et al., 2001; Kreyling, 2010). As projected by our model – and in accordance to recent publications (Wasowicz et al., 2013) – with proceeding climate change the potential suitable habitat of *L. nootkatensis* will expand significantly into the high elevation ecosystems of Iceland during the years 2061–2080, potentially due to warming and a prolonged growing seasons. In accordance to Wasowicz et al. (2013) we found human-mediation and temperature-related variables to be the most important factors shaping the distribution of *L. nootkatensis* across Iceland under current climate conditions. Wasowicz et al. (2013) interpreted this pattern as a limitation of the alien plant due to the harsh climatic conditions of Iceland. Although this explanation is probably true for most alien plant species of Iceland, it might not be applicable to the “high-latitude invader” lupine as the climate envelope of the native versus invasive range is very similar: both range from a cold temperate (boreal) to sub-polar climate (Wasowicz, 2016). Single plants and small lupine stands are not detected by the remote sensing technique used to derive our species occurrence data set, but are already recorded as present and invasive in the Icelandic highlands and mountainous areas (Wasowicz, 2016). Although the majority of lupine patches occur in the lowlands, the invader might not be limited to these climatically favorable regions close to manmade infrastructure. Our model neither confirmed a dependency of *L. nootkatensis* toward areas with high precipitation as indicated by Magnusson (2010), nor did the precipitation parameters show a high relative contribution to the Maxent model. We therefore assume that *L. nootkatensis* is already adapted to the climate of Iceland, but the predicted invasive range under the current climate conditions is biased, i.e., underestimates the potential distribution, due to the manmade distribution together with a dispersal lag of the invader. To partially exclude this bias as well as to estimate the maximum area at risk to be changed by the invader, we calculated two separate models one with and one without human infrastructure as propagation vectors.

The question arises whether the Central Highlands subsequently lose their function as a refuge for cold-adapted native species due to the projected habitat expansion and induced succession of *L. nootkatensis*. The Central Highlands and mountainous regions, especially of northern Iceland, are biodiversity hot-spots (Wasowicz et al., 2014). They are habitat to many native, cold-adapted plant species (Wasowicz et al., 2014), which are adjusted to the harsh climate (Wasowicz et al., 2013; Wasowicz, 2016) and nutrient-poor soils of arctic environments (Arnalds, 2004; Liška and Soldán, 2004; Dowdall et al., 2005). Via the accumulation of litter and atmospheric nitrogen *L. nootkatensis* eventually increases soil quality and depth (Sigurdardottir, 2008; Magnusson, 2010) and finally induces succession (Magnusson et al., 2008). Thus, the invasive ecosystem engineer pursues niche construction (Fei et al., 2014) and might act as a transformer species. In our experiments, species-rich habitats like the heathland showed a decrease in plant species diversity and richness as well as a change in community composition as soon as lupine invasion occurs, while species-poor habitats, e.g., grassland and woodland, showed an increase. There are reasons to believe that Arctic plant species probably do not tolerate elevated N as caused by lupine invasion and might be poorer competitors compared to non-native nitrophilous plants (Chapin et al., 1986; Lilleskov et al., 2002; Hofland-Zijlstra and Berendse, 2009). For example, elevated nitrogen levels lead to a decrease in the mycorrhiza community and, combined with shading, to a reduced production of phenols and tannins, resulting in a diminished competitive ability of heathland plants (Lilleskov et al., 2002; Hofland-Zijlstra and Berendse, 2009). Thus, as shown for the heath communities, a loss of plant species diversity and richness must be assumed. Additionally, elevated soil nutrients may lead to a facilitated settlement of further invasive species (Simberloff and Von Holle, 1999), which has already been demonstrated for old lupine stands (Magnusson et al., 2008; Magnusson, 2010). By altering plant community organization and by inducing succession (Appendix Figures A1, A2 and Table A1) *L. nootkatensis* changes the functional integrity of the respective habitats.

Most species will not be able to keep pace with the rapidly changing climate as their migration rates are considerably lower than the expected range shifts (Cunze et al., 2013). This is especially relevant for ecosystems in cold biomes such as Iceland, where suitable climate space is limited. On the other hand, invasive species may benefit from climate warming allowing accelerated spread. Both lead to significant changes in the native vegetation and therewith to the loss of unique ecosystems. The changes in soil properties and succession induced by lupine invasion will further speed up vegetation changes induced by climate change. It is unlikely that the native vegetation is able to adapt fast enough to those ecosystem changes.

In current as well as in future climate conditions, the amount of projected suitable habitat for *L. nootkatensis* will mainly cover areas without native vegetation (Appendix Table A2). Thus, the ecosystem engineer *L. nootkatensis* could induce the urgently needed succession to higher plant communities, which are able to stabilize the barren and sometimes degraded soils and subsequently reduce desertification and dust storms on Iceland (Arnalds and Runolfsson, 2008; Magnusson et al., 2008; Riege, 2008). However, up to 86.9% of the area currently domicile to the native vegetation of Iceland is projected to become suitable lupine habitat in future climate conditions and thus will be at risk of being permanently changed to a secondary vegetation. It is very probable that the emerging plant community differs in structure and composition from native plant communities of Iceland (Magnusson et al., 2008). The maps of the potential distribution of *L. nootkatensis* across Iceland only show the amount of projected suitable habitat, thus they give an estimate of which areas are generally endangered by lupine invasion. Those projected areas are not necessarily simultaneously covered by *L. nootkatensis* as succession might eventually lead to the displacement of the invader (Magnusson et al., 2008). However, as the emerging vegetation does not necessarily correspond to the original native vegetation of Iceland, the SDM projections predict the maximum potential area at risk to be permanently changed by *L. nootkatensis*. In addition, not only the plants, but also invertebrates and birds are affected by lupine induced homogenization (Davidsdottir et al., 2016).

## Conclusion

Invasion of an ecosystem engineer into a sub-polar environment can induce very different effects. In heavily degraded habitats it can cause a fast increase in plant species richness and diversity, while in native, cold-adapted habitats it might lead to a reduction in plant species richness by outcompeting more sensitive species. In areas where positive aspects prevail, ecosystem engineers might carefully be used for restoration purposes, e.g., to induce succession toward a stable vegetation cover on severely degraded soils. However, the spread beyond such areas is very likely leading to altered energy and nutrient fluxes. The resulting changes in ecosystem-level properties are, due to the low conversion rates of those ecosystems, long-lasting, or permanent. A change in the limiting factors, e.g., due to climate change, might lead to a massive expansion of the potential habitat, which additionally hampers the targeted application of the ecosystem engineer and facilitates invasion.

## Author Contributions

VV and AJe conceived the ideas. VV and VW designed methodology. VV, NT, AJa, VW, and PW collected and analyzed the data. VV led the writing of the manuscript. NT, AJa, VW, CB, PW, and AJe helped with the writing of the manuscript. All authors contributed critically to the drafts and gave final approval for publication.

## Conflict of Interest Statement

The authors declare that the research was conducted in the absence of any commercial or financial relationships that could be construed as a potential conflict of interest.

## Footnotes

1 http://en.ni.is/
