# Supplementary material for: Invasion of a Legume Ecosystem Engineer in a Cold Biome Alters Plant Biodiversity
Source: Front Plant Sci. 2018 Jun 5;9:715. doi: 10.3389/fpls.2018.00715 (PMC5996276; doi:10.3389/fpls.2018.00715)
Supplement: Supplementary file 1 [file Data_Sheet_1.pdf]

Appendix

Invasion of a legume ecosystem engineer in a cold biome alters plant biodiversity.

Vanessa M.S. Vetter[1,2], Nils B. Tjaden, Anja Jaeschke, Constanze Buhk, Veronika Wahl, Pawel Wasowicz, Anke Jentsch

[1]Disturbance Ecology, BayCEER, University of Bayreuth, Universitätsstr. 30, 95447 Bayreuth, Germany

[2]Biogeography, BayCEER, University of Bayreuth, Universitätsstr. 30, 95447 Bayreuth, Germany

vanessa.vetter@uni-bayreuth.de

Material and Methods

Study species

Lupinus nootkatensis (Fabaceae;) is a long-lived (up to 20 years) herbaceous plant originating from coastal regions of the Aleutian Islands and from Queen Charlotte Island, Alaska to Vancouver Island, British Columbia, Canada (Magnusson, 2010). *L. nootkatensis* was introduced to Europe in 1795 when it became a popular garden plant in England (Magnusson, 2010). From there, it reached its present distribution across Scandinavia, Iceland and Faroe islands beginning its propagation process with Sweden in the 1840s (Magnusson, 2010). In 1945, the species was introduced to Iceland from where it was spread to Greenland in 1970 and shortly after 1970 to the Faroe Islands. Nowadays *L. nootkatensis* is "not found" across large parts of Europe except for the Faroe Islands and Ireland where it is found but "not established", Finland and Sweden ("rare"), Greenland ("local"), Norway ("common") and in Iceland where it is "very common" (Magnusson, 2010). The species is mainly dispersed by human activity since it is used as an ornamental plant, to prevent soil erosion e.g. on roadsides and for land reclamation (Magnusson, 2010). The hemicryptophyte rarely produces rhizomes but is mainly spread by seeds (up to 2000 per individual with 25 flowering stems and 1800 seeds/m^2 in patches), which are generated to a large part (70 %) via self-fertilization. Lupine patches expand 1-2 meters a year as seeds are radial dispersed up to 3 meters around the mother plant by explosively opening pods (Magnusson, 2010). Sloping ground especially in combination with landslides, melting water, watercourses or strong wind falls during winter dramatically increase the species annual self-propagation (Magnusson, 2010). Additionally, seeds are spread over long distances by wildlife, e.g. birds, rivers or humans. *L. nootkatensis* produces persistent seed banks in the soil (Magnusson, 2010). *L. nootkatensis* prefers open habitats of frequent natural disturbance (Fremstad and Elven, 2008), e.g. early successional stages with vegetation destruction and soil erosion. In Iceland, the lupine is primarily recorded from gravel bars along the coast and rivers, along railways and roads, dry slopes and sandy beaches (Magnusson, 2010). But it is also found in disturbed landscapes, e.g. vegetation destruction and soil erosion, as well as in dwarf shrub-heathlands (Magnusson, 2010). According to Wasowicz et al. (2013), annual mean temperature, maximum temperature of warmest month, mean temperature of warmest quarter and mean temperature of wettest quarter might be the most important climatic factors influencing the distribution of *L. nootkatensis* in Iceland, while altitude seems not to limit the spread of the invader. While Magnusson et al. (2008) found the species to be dependent upon relatively high precipitation, this was not confirmed by the modeling approach of Wasowicz et al. (2013).

Community composition, species abundance and nearest neighbor growth patterns

Community composition was analyzed on the basis of the Londo-scale (Londo, 1976). Species abundance was measured as the relative abundance of each single species in one plot given in percentage. As, especially in the heathland, the

abundance of the different species was frequently low, all individuals of these rare species were counted. Species abundance for every species in each plot was then calculated as follows:

$$species\ abundance = \frac{\sum individuals\ of\ one\ species}{\sum individuals}$$

The mean species abundance is the relative species abundance of each species averaged over all plots within one lupine cover level.

Additionally, overall vegetation cover, moss cover and depth, lichen cover, percentage of organic material, soil depth, percentage of bare ground and the depth of the ash layer was measured or estimated per plot. We performed nearest neighbor analysis to evaluate growth patterns in lupine plots as well as facilitation and restriction of native plants. For that, one *L. nootkatensis* specimen was chosen for every plot of the category "low" or "high density" and the distances between the sprout base of the lupine and all neighboring plants within a radius of 40 cm were measured.

Statistical Analyses

Venn diagrams were used to visualize species composition change per habitats (Ritchie et al. 2015).
We performed cluster analysis with a Euclidean distance matrix of the recorded growth patterns to evaluate interspecific interactions between the invader and the native species of the different habitats and lupine cover levels (package stats). To assess the effects of the presence of *L. nootkatensis* on the species composition of the different habitats a Detrended Correspondence Analysis (DCA) was applied on the presence-absence-data of the vegetation analysis.

Calculating difference in projection

We calculated the difference in model projections by subtracting binary presence-absence maps of model 1 (with variable "distance to nearest road") from model 2 (without variable "distance to nearest road") for every time step, model and RCP respectively. Binary presence-absence maps were created using the cloglog output of the Maxent projections: all values ≥ threshold were set to 1, while all values < threshold were set to 0. We used the Maximum training sensitivity plus specificity threshold (MTSS): $MTSS_{model\ 1} = 0.531$, $MTSS_{model\ 2} = 0.553$. All calculations were done in R.

$$difference\ in\ projection = binary\ output\ (model\ 2 - model\ 1)$$

Calculation of percentage cover

In R, we calculated the amount of habitat [%] currently 1) vegetated by native Icelandic plant species and 2) barren soil which is projected to be suitable lupine habitat under present day and/or future climate conditions. First, we created two binary land cover maps with 1 = vegetation or barren soil respectively and 0 = all other land cover classes. Second, we calculated the percentage of cells shared between the binary native vegetation/binary barren soil maps and the binary presence-absence maps for every time step, model and RCP respectively.

Results and Discussion

Species list

The most diverse ecosystem in Iceland is the heathland, which accommodates 40 different species on a plot size of 2 x 2 m. Species numbers of the grassland and woodland are similarly low with six and seven species respectively. In total we found 72 different species (Tab. A1). The main difference in species richness and diversity was found to be among habitats rather than lupine cover levels.

Table A1: List of taxa recorded in the course of the vegetation analysis and their occurrence in the respective habitats and lupine cover levels. Notation of habitats and levels: YXXX: the first letter stands for the habitat [Y], all following letters determine the respective lupine cover levels the taxa was found in [XXX]. Habitat: W = woodland, H = heathland, G = grassland; lupine cover level: N = no (0 %), L = low (10 - 50 %), H = high (51 – 100 %) cover. Different habitats and their levels are separated by comma. In total 72 different taxa were distinguished.

Taxa	Habitats and levels	Taxa	Habitats and levels
Agrostis capillaris	W(NL)	*Hieracium spec.*	H(NLH), W(H)
Agrostis spec.	H(LH)	*Hieracium thaectolepium*	H(NH)
Agrostis stolonifera	G(NH), H(N), W(H)	*Juncus trifidus*	H(NL)
Agrostis vinealis	G(NL), H(NLH), W(H)	*Juncus triglumis*	H(N)
Alchemilla alpina	H(L)	*Kobresia myosuroides*	H(N)
Angelica archangelica	G(NLH)	*Lathyrus japonicus*	H(NLH), W(L)
Angelica sylvestris	G(H), H(N), W(NLH)	*Leontodon spec.*	H(N)
Anthoxanthum odoratum	H(N), W(NL)	*Listera cordata*	H(N)
Arctostaphylus uva-ursi	H(NLH)	*Lupinus nootkatensis*	G(LH), H(LH), W(LH)
Armeria maritima	H(N)	*Luzula multiflora*	G(L), H(NLH), W(NL)
Bartsia alpina	H(NL), W(N)	*Luzula spec.*	H(NLH)
Betula nana	H(NLH)	*Luzula spicata*	H(N)
Betula pubescens	G(H), H(NLH), W(NLH)	*Myosotis arvensis*	G(N)
Bistorta vivipara	H(NLH), W(NL)	*Parnassia palustris*	H(NH)
Calluna vulgaris	H(NLH)	*Pinguicula vulgaris*	H(NLH)
Campanula rotundifolia	H(NLH), W(LH)	*Platanthera hyperborea*	H(N)
Carex capillaris	H(N)	*Poa annua*	W(N)
Carex maritima	H(N)	*Poa glauca*	H(LH), W(L)
Cerastium fontanum	H(L), W(L)	*Poa pratensis*	G(NLH), W(L)
Cyperaceae	H(L)	*Poaceae*	H(NLH)
Dactylorhiza maculata	H(N)	*Potentilla crantzii*	H(NH)
Deschampsia flexuosa	G(LH), H(NLH), W(NLH)	*Ranunculus acris*	G(H), W(N)
Dryas octopetala	H(NL)	*Rhinanthus minor*	H(N)
Empetrum nigrum	H(NLH)	*Rubus saxatilis*	H(H), W(NLH)
Equisetum arvense	W(NLH)	*Rumex acetosa*	G(NLH), W(NLH)
Equisetum hyemale	W(NLH)	*Salix herbacea*	H(N)
Equisetum pratense	W(NLH)	*Salix lanata*	H(NL)
Equisetum variegatum	W(NLH)	*Salix phylicifolia*	G(L), H(N), W(NL)
Erigeron spec.	H(NLH)	*Selaginella selaginoides*	H(N)
Euphrasia frigida	H(LH)	*Silene acaulis*	H(N)
Festuca richardsonii	G(N), H(NLH), W(NLH)	*Taraxacum spec.*	G(L), W(H)
Festuca spec.	H(NLH)	*Thalictrum alpinum*	H(NLH)
Festuca vivipara	G(NL), H(NLH), W(NLH)	*Thymus arcticus*	H(NLH)
Galium normannii	H(LH), W(LH)	*Tofieldia pusilla*	H(NLH)
Galium verum	G(NL), H(NH), W(NLH)	*Trisetum triflorum*	G(L), H(NH)
Geranium sylvaticum	G(H), W(NLH)	*Vaccinium uliginosum*	H(NLH), W(NL)

Based on the Venn-Diagrams and the mean species abundance (Fig. A1), community composition changed the most in the grassland and the least in the heathland due to the presence of *L. nootkatensis*. In the grass- and woodland, lupine invasion increased the number of level-unique-species (species which are only present in one of the respective lupine cover levels). Lupine level "none" of the grassland showed a proportionally low number of species, which were not present in the other two levels, while level "low" and "high" had rather high numbers of level-unique-species (Fig. A1, a-c). In the woodland, the number of level-unique-species were rather equally distributed (Fig. A1, a-c). Level "none" in the heathland had a proportionally high number of level-unique-species, while the lupine cover levels "low" and "high" comprised a significantly lower number of level-unique-species (Fig. A1, a-c).

Heathland species composition was, compared to the woodland and grassland, not remarkably altered by the introduction of new species with advancing lupine invasion but by a relatively high species loss (Fig. A1, a – c). For typical heath species (Fig. A1, d) a slight decrease in abundance was found with a simultaneous increase in *Betula pubescens*. Woodland species showed very different changes in abundance with the invading lupine (Fig. A1 e). Remarkably, the cover of *Betula pubescens* increased with invader presence. The abundance of one of the most notable species of the woodland community *Geranium sylvaticum*, however, decreased drastically with invading lupine density while the abundance of *Angelica archangelica* increased.

However, the ordination graphic (Fig. A2) showed distinct differences of species composition with ongoing invasion in the heathland. Plots of level "high" and "low" generally reach higher values on the invasion-axis (DCA2), while plots of the level "none" were allocated to lower values on the invasion-axis.

The DCA-analyses (Fig. A2) showed that, heathland community composition endured a strong negative alteration by the invading lupine: decrease in species richness, decrease in the abundance of typical heath species, increase in tree cover (e.g. *Betula pubescens*). The plant community of the heath was reorganized: a vegetation type which is mainly characterized by dwarf growth changed to a vegetation type characterized by tall growing plant e.g. trees. Thus, *L. nootkatensis* alters the functional integrity of the heathland community.

L. nootkatensis changes the composition of the woodland shrub community as well as species abundances. For example, the abundance of *G. sylvaticum* declined and *R. acris* disappeared completely, while new nitrophilous shrub species (*Taraxacum spec*) arrived. Those effects might be related to additional shading effects as well as nitrogen enrichment of the soils invaded by the lupine. Native species of the grassland disappeared, leading to great dissimilarities between plots with and without *L. nootkatensis*. The invader transformed the plant community from a grassland to a vegetation with tall growing shrubs and small trees. Thus, the lupine induces succession.

The differences between the habitats are much more distinct than between the lupine cover levels and community composition did not converge among the three habitat types with increasing lupine cover level (Fig. A2).

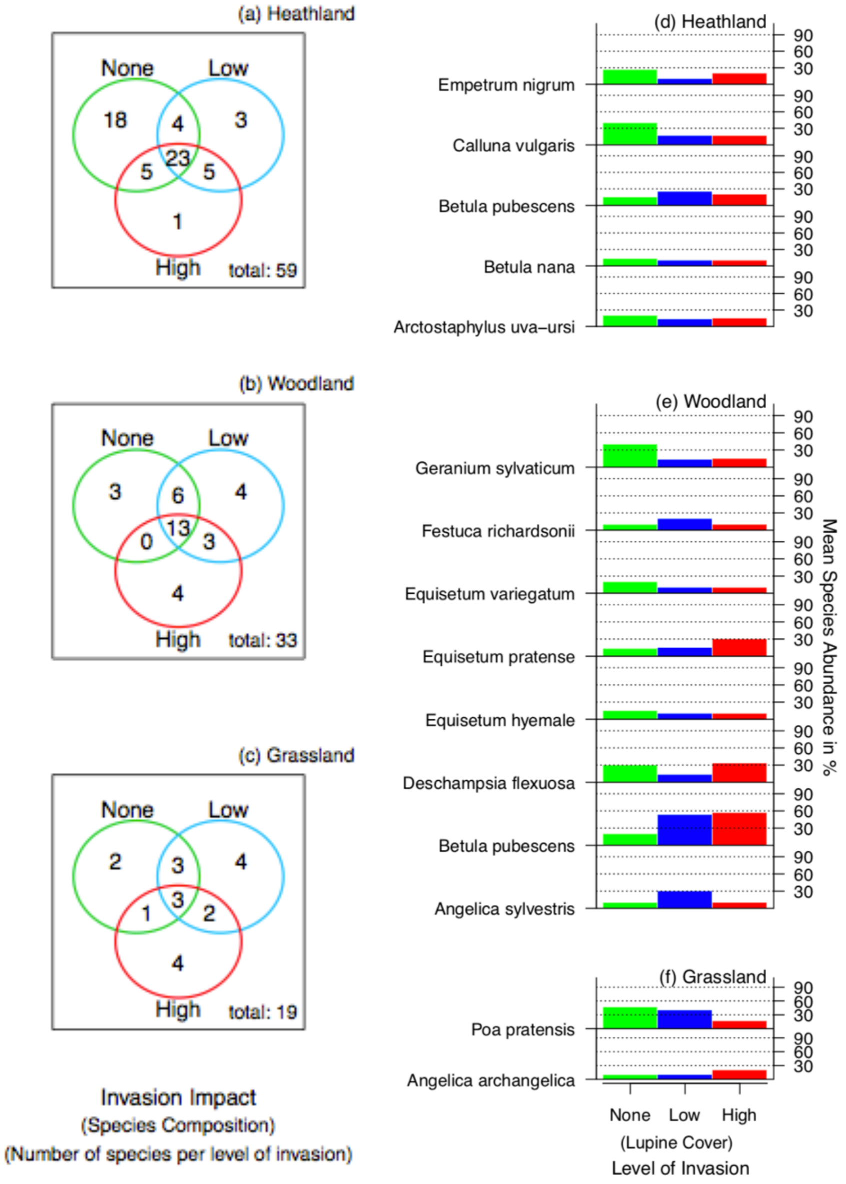

Figure A1: Impact on lupine invasion on species composition and abundance. (a – c) Venn-Diagrams visualize the impact of invasion on the species composition of the community. Number of species exclusive for or shared by the different stages of lupine invasion are indicated by the numbers in the respective (inter-)sections. (d – f) Mean abundance of species affected by different levels of lupine invasion. Level of lupine invasion was measured in the lupine cover classes “no” (0 %), “low” (< 50 %) and “high” (51-100 %).

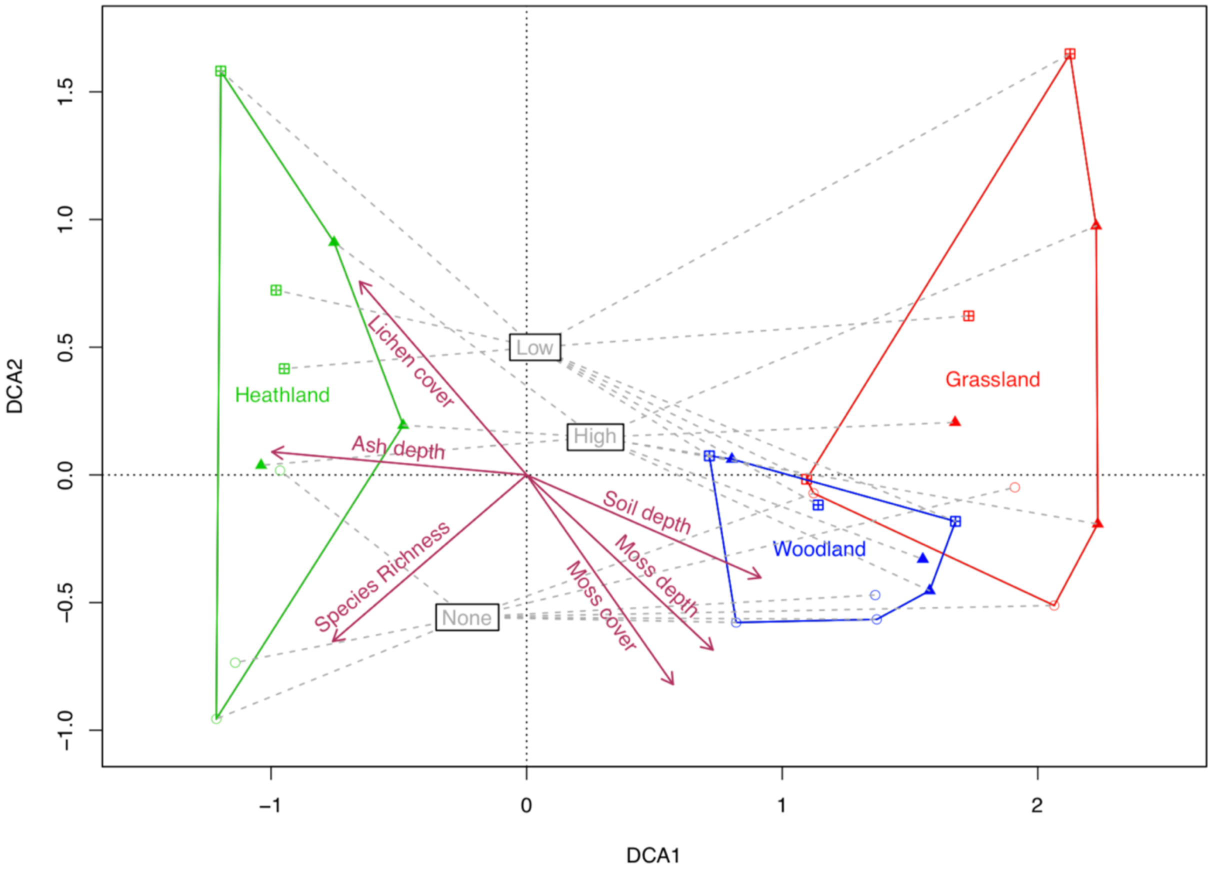

Figure A2: Detrended correspondence analysis (DCA) on presence-absence-data of the vegetation analysis including an environmental fit of background data. DCA1 = habitat-axis: differences in the species composition between the habitats; DCA2 = invasion-axis: differences among lupine cover level [“none” (0 %), “low” (10 - 50 %), “high” (51-100 %)]. Single plots of the three habitat types are displayed using different symbols in the respective color of the habitat (green: heathland, blue: woodland, red: grassland). Plots belonging to the same lupine cover level are displayed in the same symbol (circle: “none”, square: “low”, triangle: “high”) and are linked by a grey ordispider. The centroids of the ordispiders are labeled with the respective lupine cover level. The significant environmental parameters are indicated by maroon arrows.

Nearest neighbor growth pattern

Analysis of the nearest neighbor growth patterns did not indicate any vascular plant species to have interspecific interactions with *L. nootkatensis* (Fig. A2). Only bryophytes in general were able to grow closest to the stem basis of *L. nootkatensis*. In all habitats, a positive interaction between lupine and the bryophytes were indicated. In the heathland an additional indication for a positive interaction with *Calluna vulgaris* was found and a negative interaction with *Agrostis vinealis* and *Betula nana*. Indication for negative interactions with *Agrostis vinealis* and *Equisetum variegatum* were found in the woodland, while a minor positive interaction of the lupine with *Poa pratensis* was found in the grassland.

a)

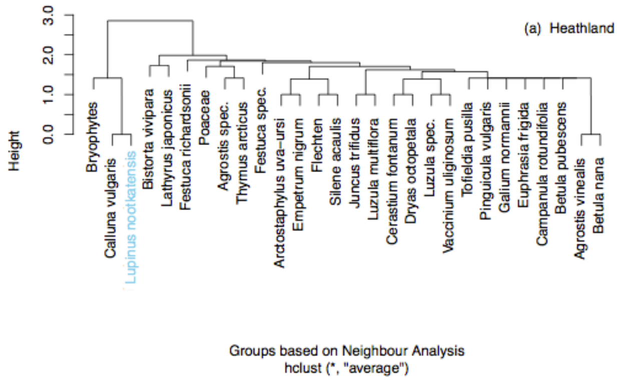
(a) Heathland
Height
0.0 1.0 2.0 3.0
Bryophytes
Calluna vulgaris
Lupinus nootkatensis
Bistorta vivipara
Lathyrus japonicus
Festuca richardsonii
Poaceae
Agrostis spec.
Thymus arcticus
Festuca spec.
Arctostaphylus uva–ursi
Empetrum nigrum
Flechten
Silene acaulis
Juncus trifidus
Luzula multiflora
Cerastium fontanum
Dryas octopetala
Luzula spec.
Vaccinium uliginosum
Tofieldia pusilla
Pinguicula vulgaris
Galium normannii
Euphrasia frigida
Campanula rotundifolia
Betula pubescens
Agrostis vinealis
Betula nana
Groups based on Neighbour Analysis
hclust (*, "average")

b)

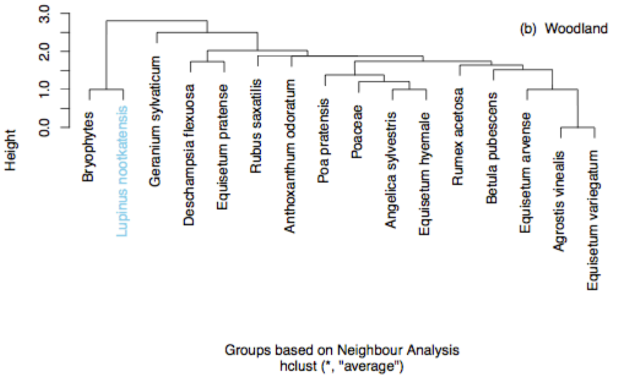
(b) Woodland
Height
0.0 1.0 2.0 3.0
Bryophytes
Lupinus nootkatensis
Geranium sylvaticum
Deschampsia flexuosa
Equisetum pratense
Rubus saxatilis
Anthoxanthum odoratum
Poa pratensis
Poaceae
Angelica sylvestris
Equisetum hyemale
Rumex acetosa
Betula pubescens
Equisetum arvense
Agrostis vinealis
Equisetum variegatum
Groups based on Neighbour Analysis
hclust (*, "average")

c)

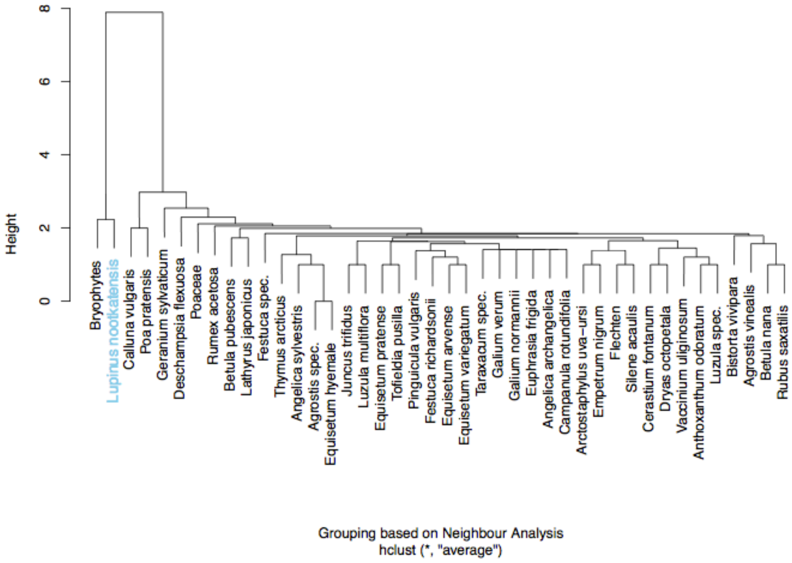
Height
0 2 4 6 8
Bryophytes
Lupinus nootkatensis
Calluna vulgaris
Poa pratensis
Geranium sylvaticum
Deschampsia flexuosa
Poaceae
Rumex acetosa
Betula pubescens
Lathyrus japonicus
Festuca spec.
Thymus arcticus
Angelica sylvestris
Agrostis spec.
Equisetum hyemale
Juncus trifidus
Luzula multiflora
Equisetum pratense
Tofieldia pusilla
Pinguicula vulgaris
Festuca richardsonii
Equisetum arvense
Equisetum variegatum
Taraxacum spec.
Galium verum
Galium normannii
Euphrasia frigida
Angelica archangelica
Campanula rotundifolia
Arctostaphylus uva–ursi
Empetrum nigrum
Flechten
Silene acaulis
Cerastium fontanum
Dryas octopetala
Vaccinium uliginosum
Anthoxanthum odoratum
Luzula spec.
Bistorta vivipara
Agrostis vinealis
Betula nana
Rubus saxatilis
Grouping based on Neighbour Analysis
hclust (*, "average")

d)

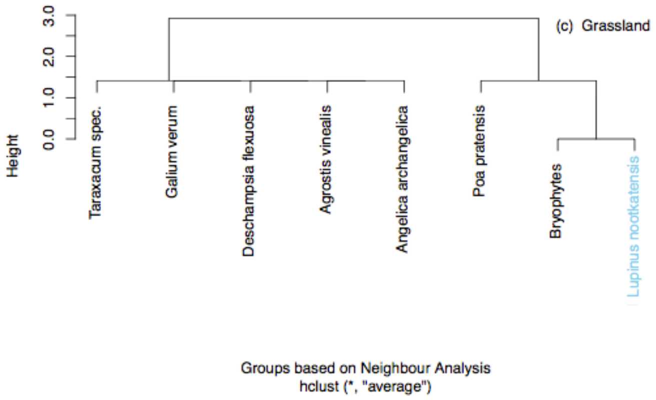
(c) Grassland
Height
0.0 1.0 2.0 3.0
Taraxacum spec.
Galium verum
Deschampsia flexuosa
Agrostis vinealis
Angelica archangelica
Poa pratensis
Bryophytes
Lupinus nootkatensis
Groups based on Neighbour Analysis
hclust (*, "average")

Figure A3: Indications for interspecific interactions with *Lupinus nootkatensis* – Cluster-analysis of the growth patterns around single lupine individuals in the primary stage of lupine invasion. Level of lupine invasion was measured in the lupine cover classes "no" (0 %), "low" (10 - 50 %) and "high" (51 - 100 %). The analysis is based on an Euclidean matrix combined with a cluster analysis with mode "average". a) No distinct indication for facilitative or inhibitive inter-habitat interactions with the lupine were found, except for the Bryophytes. b) Positive interaction with *Calluna vulgaris*; c) negative interaction with *Agrostis vinealis* and *Equisetum variegatum*; d) minor positive interaction with *Poa pratensis*. b-c) positive interaction with Bryophytes.

No indication was found that a universal vascular plant interspecific interaction existed in all three habitats. Bryophytes were able to grow close to the lupine indicating positive interactions as previously found by Magnusson et al. (2008)

Calluna vulgaris was found growing closest to the lupine in the heathland. However, this is not surprising as the *C. vulgaris* is the predominant heath species. Thus, this might not necessarily be an indication for a positive interspecific interaction, especially as the general abundance of *C. vulgaris* was greatly reduced by the invading lupine.

Species-rich habitats like the heathland show a loss of species diversity and loss in species richness as well as a change in community composition as soon as lupine invasion occurs, while species-poor habitats, e.g. grassland and woodland, show an increase. The highly competitive *L. nootkatensis* shows strong negative impacts upon small resident species – small rosettes, cushion plants and orchids – of the heathland. *L. nootkatensis* is able to outcompete native vegetation and to use the surplus of resources (Arnalds and Runolfsson, 2008). The nitrogen fixation of *L. nootkatensis* is possibly responsible for the opposing reactions of species-poor versus species-rich habitats. A slight increase in soil nitrogen of a nutrient-poor habitat, like the heathland, leads to a loss of those species, which are poor competitors and thus dependent on nutrient depletion. Elevated nutrient and especially nitrogen levels threaten the mutualistic ericoid mycorrhiza of the heath (Hofland-Zijlstra and Berendse, 2009; Lilleskov et al., 2002). This results in a loss of mycorrhiza diversity and a shift in the parasite-mutualism continuum between plant and fungi; both affects the aboveground vegetation (Hofland-Zijlstra and Berendse, 2009; Lilleskov et al., 2002). An increase of soil nitrogen availability combined with shading, furthermore decreases the production of phenols and tannins in heath, resulting in a reduction of the competitive ability of heathland plants (Hofland-Zijlstra and Berendse, 2009). Nitrogen enrichment in the soil of species-poor grass- and woodlands leads to a slight increase in the total number of species, while a drastic increase of soil nitrogen caused by high lupine covers leads to the loss of specialist species and to a replacement by widespread nitrogen-using plants (e.g. *Taraxacum spec.* in the woodland or *Geranium sylvaticum* in the grassland) and late successional species (e.g. *Betula pubescens* and *Angelica archangelica*). Tall nitrophilous plants outcompete small resident species of open habitats, *e.g. Salix herbacea*, via competition for light (Mueller et al., 2016). By altering plant community organization and by inducing succession (Tab. A1, Fig. A1 – A2) *L. nootkatensis* changes the functional integrity of the respective habitats.

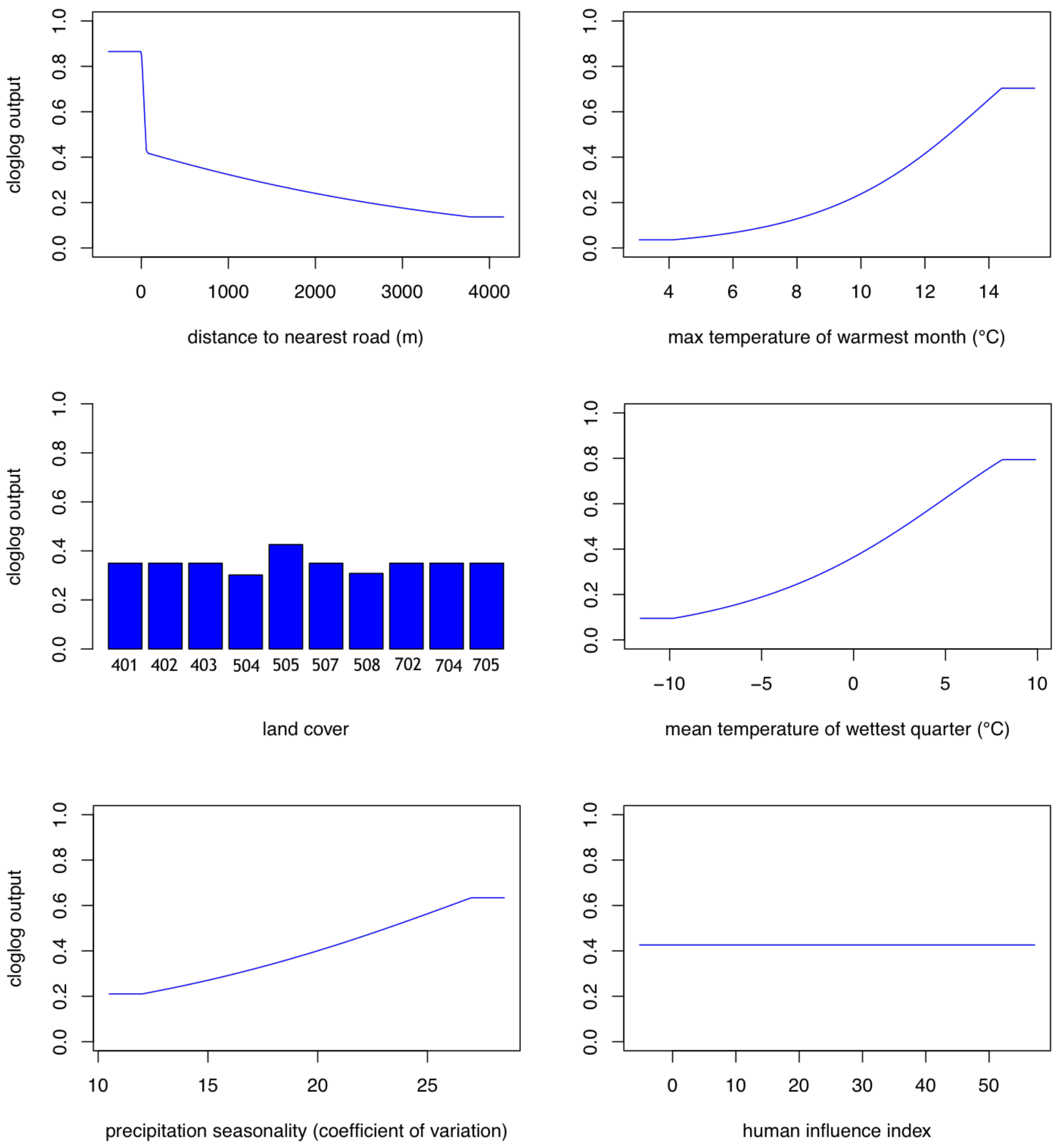

Figure A4: Marginal response curves of the environmental variables used to fit the final Maxent model 1. These response curves are fitted individually for each environmental variable, while the other predictors are kept at their average value. Environmental suitability (or the predicted probability of presence of the modeled species) ranges from: minimum = 0 to maximum = 1. Land cover categories: 401 = sand, 402 = lava, 403 = gravel plain, 504 = moss heath, 505 = grassland, heath, cultivated land, 507 = Birch woodland, 508 = wetland, 702 = rivers, 704 = water bodies, 705 = glaciers.

Table A2: Proportion of shared habitat between *L. nootkatensis* and the native vegetation of Iceland as well as proportion of bare soil projected to be covered by *L. nootkantensis* within the different time scales. Total area of Iceland covered by native vegetation: 45.9%, total area covered by bare soil: 41.3%.

Time scale	Concentration pathway	Total area covered by *L. nootkatensis* [%]	Proportion of covered native vegetation	Proportion of covered bare soil
**current**	**-**	13.3	24.1	26.8
**MPI_ESM-LR**	**RCP 4.5**	39.1	61.3	68.0
**MPI_ESM-LR**	**RCP 8.5**	61.7	86.9	96.4
**NorESM1-M**	**RCP 4.5**	50.1	74.5	82.6
**NorESM1-M**	**RCP 8.5**	58.0	81.3	90.2

In current as well as in future climate conditions, the amount of projected suitable habitat for *L. nootkatensis* will mainly cover areas without native vegetation (Tab. A2). However, in future climate conditions, up to 86.9% of the area currently domicile to the native vegetation of Iceland is projected to become suitable lupine habitat and thus 87% of the vegetation ecosystems of Iceland will be at risk of being permanently changed to a secondary vegetation due the invasive ecosystem engineer. In addition, 96.4% of the currently vegetation-free areas of Iceland were projected to become suitable lupine habitat in future climate conditions. This might eventually lead to a massive transformation of the majority of Icelandic ecosystems, especially under the assumption that nowadays barren soils (especially in the high elevation ecosystems) might become important refuges for native species in the context of climate change. However, if these high altitudinal refuges have already been altered by *L. nootkatensis*, colonization of indigenous species might no longer be possible. Subsequently, this could lead to the accelerated extinction of indigenous species in the context of climate change.

References

Arnalds, A., and Runolfsson, S. (2008). "The role of Nootka Lupin (Lupinus nootkatensis) for revegetation in Iceland," in *Lupins for health and wealth: 12th International Lupin Conference, Fremantle, Western Australia, 14-18 September 2008*, eds. J. A. Palta and J. D. Berger (Canterbury, New Zealand: International Lupin Association).

Fremstad, E., and Elven, R. (2008). "Perennial Lupins in Fennoscandia," in *Lupins for health and wealth: 12th International Lupin Conference, Fremantle, Western Australia, 14-18 September 2008*, eds. J. A. Palta and J. D. Berger (Canterbury, New Zealand: International Lupin Association).

Hofland-Zijlstra, J. D., and Berendse, F. (2009). The effect of nutrient supply and light intensity on tannins and mycorrhizal colonisation in Dutch heathland ecosystems. *Plant Ecol.* 201, 661–675. doi:10.1007/s11258-008-9554-3.

Lilleskov, E. A., Fahey, T. J., Horton, T. R., and Lovett, G. M. (2002). Belowground Ectomycorrhizal Fungal Community Change Over a Nitrogen Deposition Gradient in Alaska. *Ecology* 83, 104. doi:10.2307/2680124.

Londo, G. (1976). The decimal scale for relevés of permanent quadrats. *Plant Ecol.* 33, 61–64.

Magnusson, B. (2010). NOBANIS - Invasive Alien Species Fact Sheet - Lupinus nootkatensis.

Magnusson B., Magnusson S.H., and Sigurdsson B.D. (2008). "Plant succession in areas colonized by the introduced Nootka Lupin in Iceland," in *Lupins for health and wealth: 12th International Lupin Conference, Fremantle, Western Australia, 14-18 September 2008*, eds. J. A. Palta and J. D. Berger (Canterbury, New Zealand: International Lupin Association).

Mueller, I. B., Buhk, C., Lange, D., Entling, M. H., and Schirmel, J. (2016). Contrasting effects of irrigation and fertilization on plant diversity in hay meadows. *Basic Appl. Ecol.* 17, 576–585. doi:10.1016/j.baae.2016.04.008.

Ritchie, M. E., Phipson, B., Wu, D., Hu, Y., Law, C. W., Shi, W., et al. (2015). *limma powers differential expression analyses for RNA-sequencing and microarray studies.*

Wasowicz, P., Przedpelska-Wasowicz, E. M., and Kristinsson, H. (2013). Alien vascular plants in Iceland: Diversity, spatial patterns, temporal trends, and the impact of climate change. *Flora - Morphol. Distrib. Funct. Ecol. Plants* 208, 648–673. doi:10.1016/j.flora.2013.09.009.
